# Complete Annotated Genome Sequences of Two Novel Microbacteriophages, Gingerbug and HerculesXL, from Western Oregon, USA

**DOI:** 10.1128/mra.00919-22

**Published:** 2022-10-26

**Authors:** Matthew R. Fisher, Chyanna G. Blackburn, Hope T. Poet, Rebecca Meisner

**Affiliations:** a Biology Department, Oregon Coast Community College, Newport, Oregon, USA; Queens College CUNY

## Abstract

Bacteriophages Gingerbug and HerculesXL are siphoviruses that were isolated from soil in western Oregon, USA, using the actinobacterium Microbacterium foliorum. The genomes of Gingerbug and HerculesXL are similar in length and, based on gene content similarity to actinobacteriophages, were assigned to phage clusters GF and EA11, respectively.

## ANNOUNCEMENT

Discovering new bacteriophages increases our understanding of their diversity, evolution, and interactions with hosts (1). Here, we report the complete genome sequences of two novel phages, Gingerbug and HerculesXL, that infect the Gram-positive bacterium Microbacterium foliorum NRRL B-24224.

**FIG 1:**
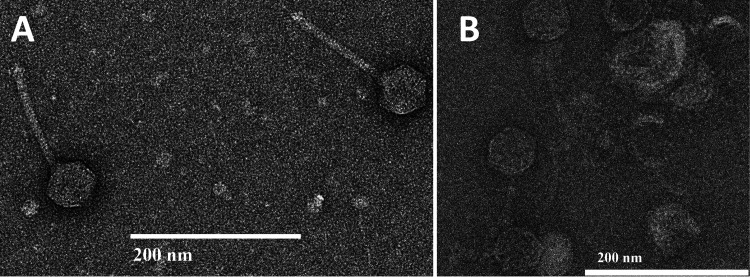
Negative staining (1% uranyl acetate) transmission electron micrographs for Gingerbug (capsid diameter, 52 to 54 nm; tail length, 123 to 128 nm [*n* = 4]) (A) and HerculesXL (B). Micrographs for HerculesXL were of insufficient quality for precise measurements.

Both phages were isolated using standard protocols (2). Soil samples (Table 1) were washed in peptone-yeast extract-calcium (PYCa) liquid medium. The wash was collected by centrifugation and filtration (0.22-μm pores), and the filtrate was inoculated with M. foliorum and cultured at 30°C for 2 to 5 days with shaking at 150 rpm. The culture was then filtered and plated in PYCa top agar with M. foliorum, and plates were incubated for 2 to 3 days at 30°C, yielding phages Gingerbug and HerculesXL. Both phages were purified with at least three rounds of plating. Negative-staining transmission electron microscopy revealed both phages to be siphoviruses (Fig. 1).

**TABLE 1:** Genome characteristics and locations of origin for the microbacteriophages Gingerbug and HerculesXL, both collected in October 2021

Phage	Genome size (bp)	G+C content (%)	No. of genes with putative functions	Total no. of genes	Cluster assignment	Soil sampling site coordinates
Gingerbug	39,721	69.3	32	69	GF	44.60271N, 124.04604W
HerculesXL	39,410	63.9	25	60	EA (subcluster 11)	44.55003N, 123.26578W

For each phage, double-stranded DNA was extracted from a high-titer lysate using the Promega Wizard DNA cleanup kit, prepared for sequencing using the NEBNext Ultra II FS kit, and sequenced using an Illumina MiSeq sequencer (v3 reagents). For Gingerbug, this produced 375,027 single-end 150-bp reads, which represented 1,343× average genome coverage. For HerculesXL, sequencing produced 515,560 single-end 150-bp reads, which represented 1,853× average genome coverage. For both phages, assembly was performed using Newbler v2.9 (3) and checked for completeness and characteristics of genome termini using Consed v29 (4). Both phages have circularly permuted genomes. Genome lengths and G+C contents are provided in Table 1.

We determined the open reading frames in each phage genome by running autoannotation in DNA Master v5.23.6 (http://cobamide2.bio.pitt.edu), which relies on Glimmer v3.02 (5) and GeneMark v2.5 (6). We then conducted manual review to revise the start sites and determine putative gene functions using BLAST (7) searches against the NCBI nonredundant database and the Actinobacteriophage Database, and HHpred (8) searches against the PDB mmCIF70, NCBI Conserved Domain, Pfam-A, and UniProt/Swiss-Prot databases, as well as Phamerator v3.0 (9), SOSUI (10), DeepTMHMM (11), and Starterator (http://phages.wustl.edu/starterator). We searched for tRNA genes using ARAGON v1.2.38 (12) but found none. All software applications were run using default parameters.

The genome of Gingerbug contains 69 protein-coding genes, of which 32 were assigned a putative function. Seven Gingerbug genes have no homologs in the Actinobacteriophage Database (13). The genome of HerculesXL includes 60 protein-coding genes, of which 4 have no actinobacteriophage homologs and 25 were assigned a putative function (Table 1). The left halves of both genomes contain rightward-transcribed structural, assembly, and lysis genes, including a tail assembly chaperone gene with a −1 programmed translational frameshift. DNA metabolism genes are distributed across the right arms of both genomes, which contain both rightward- and leftward-transcribed genes. Based on gene content similarity of at least 35% to phages in the Actinobacteriophage Database, Gingerbug and HerculesXL were assigned to phage clusters GF and EA11, respectively (14). Neither immunity repressor nor integrase functions could be identified for either phage, suggesting that they are lytic phages.

### Data availability.

The genome sequence for Gingerbug is available in GenBank with accession no. ON970592 and Sequence Read Archive (SRA) accession no. SRR20748624. The genome sequence for HerculesXL is available in GenBank with accession no. OP068330 and SRA accession no. SRR20748623.
